# Revisiting the Plausibility Effect in Remembering Truth and Falsity: An Analysis of Underlying Memory and Guessing Processes

**DOI:** 10.5334/joc.459

**Published:** 2025-09-02

**Authors:** Daria Ford, Lena Nadarevic

**Affiliations:** 1 Department of Psychology, School of Social Sciences, University of Mannheim, Mannheim, Germany; 2 Department of Psychology, Charlotte Fresenius Hochschule, Wiesbaden, Germany

**Keywords:** plausibility, veracity, feedback memory, item memory, expectation-violation model

## Abstract

Plausibility seems to play a key role in how well people remember the veracity of information. In a study by Vorms and colleagues (2022), an interaction pattern between statement plausibility and veracity feedback on memory performance appeared: Plausible statements were significantly more often correctly identified as true than correctly identified as false; for implausible statements, the descriptive trend was reversed. Given the importance of accurate memory for truth and falsity in real-world settings, it is crucial to understand the cognitive processes underlying this plausibility effect. For this purpose, we conducted a preregistered experiment in which participants studied four different statement types along with veracity feedback: plausible true, plausible false, implausible true, and implausible false. In a later recognition test, they indicated whether a statement was presented and, if so, what veracity feedback was displayed. We replicated the plausibility effect as an interaction between statement plausibility and veracity feedback on correct true/false attributions. Moreover, we analysed the data with a multinomial model to estimate the contribution of statement memory, feedback memory, and different guessing processes underlying the observable responses. These analyses revealed that guessing processes and statement memory accounted for the above-mentioned plausibility effect: Feedback guessing was influenced by corresponding statement plausibility, and statement memory was overall better when the veracity feedback aligned with statement plausibility. In contrast, feedback memory was enhanced in the case of a discrepancy between veracity feedback and statement plausibility. These results emphasise the importance of examining the processes driving the plausibility effect to derive correct conclusions.

## Introduction

When encountering information, people have a natural need to validate it; we simply want to know if the presented information (news headline, social media post, etc.) is true or false. The question remains: what happens when information is completely new and people have no prior knowledge? Research shows that the validation process occurs immediately and involuntarily (Richter, 2015; Singer, 2013, 2019; but see Wiswede et al., 2013), typically in the form of plausibility assessments of the encountered information. However, plausibility not only plays an important role in judging the veracity of information (e.g., Fazio et al., 2019), but it has also been linked to memory for truth and falsity (Niedziałkowska & Nieznański, 2021; Vorms et al., 2022). Consider the following example: when we first hear that dolphins are mammals, this is typically hard to believe as dolphins live in the sea and look more like fish than like other mammals. Nevertheless, we learn that this information is true. But does a mismatch between plausibility and actual veracity hinder or promote the memorisation of truth and falsity? Or does plausibility play no role at all?

Vorms and colleagues (2022) found that statement plausibility affected people’s correct recognition of veracity feedback (“true” vs. “false”). Specifically, plausible statements were more often correctly recognised as true than false, whereas implausible statements showed the opposite trend (albeit to a lesser extent). However, the cognitive processes that underlie this plausibility effect are not well understood. To close this research gap, we conducted a preregistered experiment building on previous experimental paradigms (Niedziałkowska & Nieznański, 2021; Vorms et al., 2022) combined with the methodological approach of multinomial modelling. To this end, we investigated the importance of statement plausibility in remembering veracity feedback and tested if we could replicate the interaction of statement plausibility and veracity feedback on correct true/false attributions as reported by Vorms and colleagues. Moreover, we disentangled statement memory, feedback memory, and guessing processes using a multinomial processing tree model to better understand the cognitive processes underlying the plausibility effect.

## Memory for veracity feedback

Various studies show that people are not as gullible as we might think (for a review, see Mercier, 2017). However, little is known about whether any systematic biases affect people’s memory for the veracity of information. The literature presents an inconsistent pattern of findings on this subject. Most studies either show that people remember truth and falsity of information at a similar level (e.g., Nadarevic, 2025, Experiment 1; Nadarevic & Bell, 2024; Nadarevic & Erdfelder, 2013, 2019, Experiment 1), or that people remember truth better than falsity (e.g., Ford & Nieznański, 2023; Nadarevic, 2025, Experiments 2 and 3; Nadarevic & Erdfelder, 2019, Experiment 2; Niedziałkowska & Nieznański, 2021). Yet, it is unclear what drives the different findings.

A study by Vorms and colleagues (2022) investigated statement plausibility—defined as a priori believability in a novel statement without prior knowledge—as a possible determinant of memory for truth and falsity.^1^ More specifically, participants saw individual statements of high versus low plausibility together with veracity feedback (“true” vs. “false” vs. no feedback) in a study phase. When assessing participants’ feedback memory in a later test, the authors observed the following interaction of statement plausibility and veracity feedback on correct feedback identifications: for plausible statements, participants were more likely to correctly identify “true” feedback than “false” feedback, whereas the opposite tendency was found for implausible statements. Interestingly, a similar interaction pattern was observed by Nadarevic and Bell (2024), who investigated the role of context-based expectations on memory for truth and falsity. In a context with a high base rate of allegedly true statements, participants were more likely to correctly identify “true” feedback than “false” feedback, whereas the opposite pattern was found in a context with a high base rate of allegedly false statements. However, using a multinomial-modelling approach, the authors found this pattern to reflect expectation-consistent guessing behavior, not memory processes. The same might therefore apply to the plausibility effect described above.

## The expectation-violation model

In their study, Nadarevic and Bell (2024) tested the expectation-violation account as a theoretical model for the memory representation of truth and falsity. The model assumes that memory for truth and falsity varies as a function of people’s veracity expectations. Specifically, the model predicts a memory advantage for the expectation-inconsistent veracity feedback. This model thus closely relates to cognitive phenomena such as schema-based expectancy (Bayen et al., 2000; Bayen & Kuhlmann, 2011; Wulff et al., 2021) or prediction error (Krawczyk et al., 2017). To test the expectation-violation model, Nadarevic and Bell induced an expectation for a specific feedback type by exposing participants to a high base rate of either allegedly “true” or “false” statements in the study phase, all of which were of moderate plausibility. However, unlike predicted by the model, the authors did not find an effect of this base-rate manipulation on feedback memory. Yet, participants showed an expectation-violation effect on statement memory (i.e., better memory for statements that were paired with the unexpected veracity feedback) and an expectation-consistent guessing bias (i.e., a higher guessing probability for the expected as compared to the unexpected veracity feedback).

Although Nadarevic and Bell (2024) could not find an expectation-violation effect on memory for veracity feedback in their study, it is possible that manipulating item-level expectations rather than context-level expectations could induce the predicted effect. More specifically, it is reasonable to assume that when people encounter plausible information, but learn that it is actually false, memory for the feedback will be better compared to expectation-consistent cases. The same assumption holds for implausible information presented with “true” feedback. This prediction is also in line with the results of Fazio and Marsh (2009) where surprising feedback (inconsistent with what is predicted) improved context memory, and—when sources were highly distinct—item memory. Building on these results, Vorms and colleagues (2022) articulated the following assumptions for the effect of plausibility-inconsistent veracity feedback: “(…) the surprise caused by the learning signal may enhance participants’ memorisation of statements and their truth-value. But, when participants do not correctly remember statements, their answer should reflect prior plausibility rather than default acceptance.” (p.4). In other words, the authors predicted an expectation-violation effect on both statement memory and feedback memory. Moreover, they assumed an expectation-consistent guessing bias. However, as the data was analysed at the level of observed responses only, the validity of these predictions remains to be tested. The aim of our preregistered experiment was to address this research gap by investigating the cognitive processes underlying the plausibility effect using a multinomial processing tree (MPT) model.

## The employed MPT model

**Figure 1:**
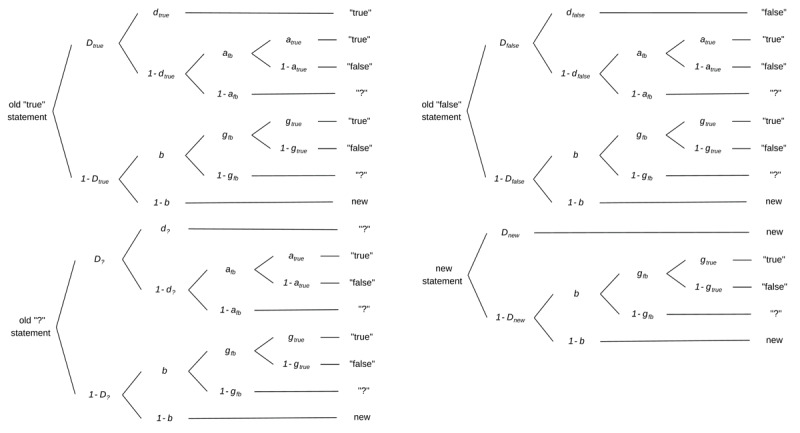
The three-sources variant of the two-high-threshold model. *Note*. The four processing trees refer to different statement conditions while branches represent cognitive processes underlying a particular response (“true”, “false”, “?”, “new”). The model’s parameters reflect item memory (*D*), feedback memory (*d*), and different guessing processes (*b, a*, and *g*).

MPT models are stochastic models that allow to disentangle and to measure the contribution of different latent cognitive processes underlying observed responses (for reviews see Batchelder & Riefer, 1999; Erdfelder et al., 2009; for a tutorial see Schmidt et al., 2025). A frequently used and extensively validated MPT source monitoring model by Bayen et al. (1996) allows to estimate parameters for item memory, source memory, and guessing processes. For our data analyses, we used a variant of the model adapted to a memory paradigm with three sources or feedback conditions, respectively (Keefe et al., 2002; see also Bell et al., 2010; Nadarevic & Erdfelder, 2013). In line with Vorms and colleagues (2022), the feedback conditions in our experiment were “true”, “false”, and no feedback. In the latter condition, we displayed a question mark (“?”) instead of veracity feedback to keep trials comparable between all feedback conditions. Figure 1 displays the respective MPT model for our experimental paradigm.

The model predicts that a statement presented with “true” feedback in the study phase will be recognised as old in the memory test with the probability *D*_true_. Thus, parameter *D* represents statement memory in the current paradigm. When participants don’t recognise the statement (probability 1 – *D*_true_), they either guess that it is old (probability *b*) or that it is new (probability 1 – *b*). When participants guess “old”, they also guess whether they received veracity feedback (probability *g*_fb_), and if so, whether this feedback was “true” (probability *g*_true_) or “false” (probability 1 – *g*_true_). In contrast, if participants guess that they have not received veracity feedback (probability 1 – *g*_fb_), they will respond “?”. In case a statement is recognised as “old”, participants also remember the “true” feedback with the probability *d*_true_. Thus, parameter *d* represents feedback memory in the current paradigm. When the feedback is not remembered (probability 1 – *d*_true_), guessing determines participants’ responses: again, participants either guess that they received veracity feedback (probability *a*_fb_) or not (probability 1 – *a*_fb_). In the former case, they also guess whether the veracity feedback was “true” (probability *a*_true_) or “false” (probability 1 – *a*_true_), whereas in the latter case, they will respond “?”. Analogically to model trees for statements presented with “true” feedback, the model also involves trees for statements presented with the feedback “false”, for statements without veracity feedback (i.e., “?”), and for new statements. In the current study, all model parameters were estimated separately for plausible and implausible statements.

## The current experiment

The experiment aimed to investigate the role of plausibility in remembering truth and falsity. Similar to Vorms et al. (2022), we presented participants with plausible and implausible statements that were either followed by “true” feedback, “false” feedback, or no veracity feedback (“?”). Unlike in the original study, in which participants had to memorise random sequences of five digits while processing the veracity feedback, we did not employ cognitive load as we aimed to investigate memory for truth and falsity in a more natural setting.^2^ Memory for the statements and the corresponding feedback was assessed in a later test. Based on the observable responses in this test, we examined the replicability and robustness of the plausibility effect reported by Vorms and colleagues characterised by an interaction of statement plausibility and veracity feedback on correct feedback attributions. Regardless of whether we would find this interaction effect, we also examined the influence of statement plausibility on statement memory, feedback memory, and guessing processes measured by the MPT model introduced above.^3^

Based on the predictions of the expectation-violation model, the findings of Nadarevic and Bell (2024), and the assumptions of Vorms et al. (2022), we tested the following hypotheses: 1) We hypothesised that statements presented along with expectation-inconsistent feedback (implausible statements with “true” feedback; plausible statements with “false” feedback) will be remembered better than statements presented along with expectation-consistent feedback (plausible statements with “true” feedback; implausible statements with “false” feedback). We tested this hypothesis by comparing the *D*-parameters for expectation-consistent and expectation-inconsistent feedback within plausibility conditions. 2) Although not found by Nadarevic and Bell (2024) when examining context-based expectations, we expected an analogous expectation-violation effect on feedback memory. This is because plausibility-inconsistent feedback should elicit stronger expectation violations at the item level than a context-based manipulation. We tested for the predicted expectation-violation effect on feedback memory by comparing the *d*-parameters for statements presented along with expectation-consistent and expectation-inconsistent feedback within plausibility conditions. 3) We hypothesised that statement plausibility would affect the probability of guessing that a statement was presented with “true” feedback. We tested this assumption by comparing the MPT models’ *a*_true_ and *g*_true_ parameters, respectively, between plausible and implausible statements.

## Methods

### Materials

We pretested 120 statements for plausibility and prior knowledge. All statements referred to the common theme of “laws, customs, and facts about countries” to avoid a confound between statement topic and plausibility. Part of the statements were adapted from Vorms and colleagues (2022) and part were created using information from various websites e.g., *Wikipedia, Statista, National Geographic*. Forty pretest participants judged the plausibility of the statements on a scale from 0 = “highly implausible” to 10 = “highly plausible”. For each statement, participants also indicated whether they knew the statement’s veracity (response options: “know – true”, “don’t know” or “know – false”). We then selected 24 statements (12 true, 12 false) with high plausibility ratings (*M* = 7.1) and 24 statements (12 true, 12 false) with low plausibility ratings (*M* = 4.1) to serve as stimuli for our experiment. Additionally, we chose 12 statements (6 true, 6 false) of medium plausibility (*M* = 5.8) to serve as buffer statements. All selected statements were characterised by little prior knowledge (proportion of correct “know” judgments <.20). The final materials and a more detailed description of the selection process are available in an online supplement on the Open Science Framework (OSF, materials: https://osf.io/rd67q, description: https://osf.io/9jnf5).

### Procedure

The experiment was programmed in lab.js https://lab.js.org/ (Henninger et al., 2024) and hosted on a JATOS server (Lange et al., 2015).^4^ The experiment consisted of three phases: a study phase, a short retention interval, and a test phase. In the study phase, participants were presented with 48 statements: six primacy buffer statements, 36 target statements appearing in random order, and six recency buffer statements. The target statements fell into one of four categories, each comprising nine statements: plausible and true (e.g. *Iran bans women from solo singing in mixed or male audiences*), plausible and false (e.g. *Dubai is the city with the most skyscrapers*), implausible and true (e.g. *In France, you can marry a dead person*), and implausible and false (e.g. *Flushing the toilet at night is illegal in Switzerland*). Individual statements were presented for 4 seconds, followed by the feedback displayed for 1.5 seconds, informing participants about the statement’s veracity (e.g. *In France, you can marry a dead person. TRUE*). Of the 18 plausible target statements, six randomly selected true statements were displayed with “true” feedback, six randomly selected false statements with “false” feedback, and the remaining six statements (three true, three false) with “?” feedback. The same held for the 18 implausible target statements. Following the study phase, there was a short retention interval in which participants had to judge the correctness of 20 mathematical equations displayed one after the other on the screen. In the test phase, participants were again presented with a set of 48 statements (36 old, and 12 new ones). For each statement, participants were asked if the statement was old or new. When participants indicated that it was old, they were also asked if the feedback displayed was “true”, “false”, or “?”. The order of response options displayed on the screen in the statement recognition task (old–new vs. new–old) and the feedback recognition task (true–false–? vs. false–true–?) was counterbalanced between participants. At the end of the experiment, participants had to indicate whether they a) worked seriously on the study, b) were distracted during the study phase, and c) wrote down information from the study phase.

### Design

The experimental design was a within-within subject design with manipulation of statement plausibility (plausible vs. implausible) and feedback condition (“true” vs. “false” vs. “?”). The dependent variable was the conditional source identification measure (CSIM, Murnane & Bayen, 1996), which is a measure of participants’ feedback attributions, defined as the proportion of correct feedback attributions among the correct “old” responses. CSIMs were calculated separately for each combination of statement type (plausible and implausible) and veracity feedback (“true”, “false”, none). Dependent variables were also the parameters of the MPT model, in particular the parameters representing statement memory (*D*), feedback memory (*d*), and a guessing tendency for “true” vs. “false” feedback (*a*_true_ and *g*_true_).

### Participants

We collected data from 398 Prolific workers based in the US since our materials had been normed on a US sample. Data of 70 participants, who met at least one exclusion criterion listed below, were excluded from analyses and replaced by new Prolific workers until the target sample size of *N* = 328 was reached. For a detailed description of the data collection process, see the laboratory log on OSF (https://osf.io/fty59). Our pre-screening criteria on Prolific were English as a first language, age between 18–40 years, and an approval rate of at least 95%. Participants provided informed consent to participate and were paid £3.50 for completing the study with a median completion time of 23 minutes.

#### Power analysis

For the analysis of CSIMs, we performed an a priori power analysis with G*Power (Faul et al., 2007) based on the following parameters. We defined *f* = .10 to be our smallest effect size of interest for the expected within-within interaction of statement plausibility (plausible vs. implausible) and veracity feedback (true vs. false). Further, we set the assumed repeated-measures correlation to ρ = .50, the target power to 1 – β = .90, and the significance level to α = .05. This power analysis indicated a minimum sample size of *N* = 135 participants. We also ran a power analysis for the planned model-based tests with the program multiTree (Moshagen, 2010). Again, we set the target power to 1 – β = .90, and the significance level to α = .05. Estimates of population parameter values required for this analysis were inferred from parameter estimates from previous studies on memory for truth and falsity (Nadarevic, 2025). The power analysis indicated a required sample size of *N* = 328 participants to detect the predicted expectation-violation effect on *d*-parameters of size Δ*d* = .10. Because the required sample sizes for the planned comparisons on the *D*-parameters and *a*_true_*/g*_true_-parameters were considerably lower, we set *N* = 328 as our target sample size. For a more detailed description of the power analyses, see the online supplement on OSF (https://osf.io/wn8vp/).

#### Criteria for data inclusion and exclusion

We excluded the data of participants when their native language was not English or they did not have very good English skills (*n* = 15), when they indicated that they did not participate seriously (*n* = 2) or took notes during participation (*n* = 32), or when they did not show any memory for the presented statements as indicated by a discrimination index *P_r_* (calculated as hit rate minus false alarm rate, Snodgrass & Corwin, 1988) equal or smaller than zero (*n* = 21). Of the final *N* = 328 participants, 179 identified themselves as male, 140 as female, eight as non-binary, and one person did not disclose their gender. The mean age of the final sample was *M* = 30.6 (*SD* = 5.6) years.

## Results

All data analyses were conducted with R (R Core Team, 2022). The data and analyses scripts are provided on OSF (demographic analyses: https://osf.io/ag3dt, analyses of feedback attributions: https://osf.io/qa2zy, multinomial-model analyses: https://osf.io/ch2p6). For the following analyses, we set the significance level to α = .05, unless stated otherwise.

### Analyses of correct feedback attributions

#### Preregistered analysis of CSIMs

**Figure 2:**
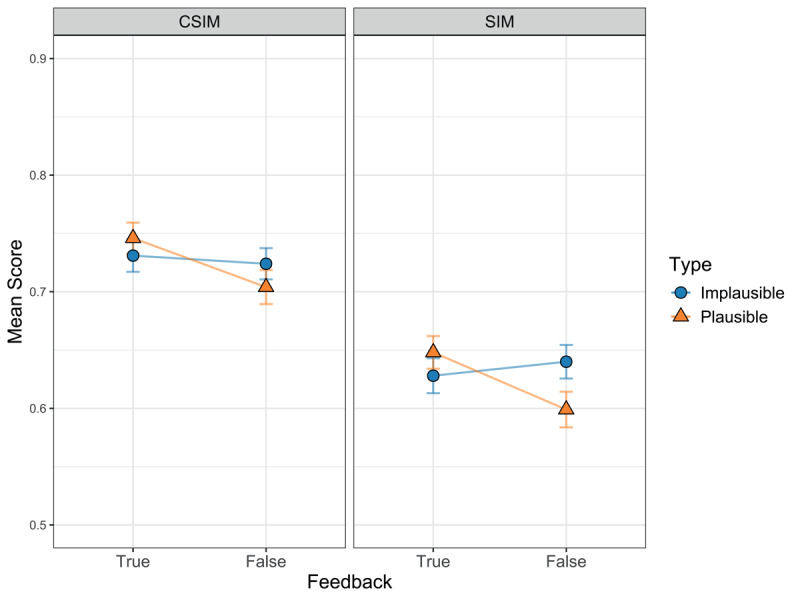
Mean scores (SE) for the Conditional Source/Feedback Identification Measure (CSIM) and the Source/Feedback Identification Measure (SIM), depending on statement type (plausible vs. implausible) and veracity feedback (true vs. false).

In line with similar previous studies (Nadarevic & Bell, 2024; Nadarevic & Erdfelder, 2013, 2019), we computed the CSIM—the proportion of correct feedback attributions among the correct “old” responses—as a measure of correct feedback attributions. We then compared participants’ CSIMs as a function of statement plausibility and veracity feedback by means of a 2 (plausibility: plausible vs. implausible) × 2 (feedback: “true” vs. “false”) repeated measures ANOVA. Three participants had to be excluded from this analysis due to missing data in at least one cell of the ANOVA design. Importantly, we expected to find an interaction between plausibility and veracity feedback and thus to replicate the results of Vorms and colleagues (2022). Although the descriptive data displayed an interaction pattern (see Figure 2, left panel), our analysis of CSIMs did not confirm a statistically significant interaction effect of statement plausibility and veracity feedback, *F*(1, 324) = 2.25, *p* = .135, η*_p_*^2^ = .007. Moreover, CSIMs did not significantly differ between plausible statements (*M*_plausible_ = .73) and implausible statements (*M*_implausible_ = .73), *F*(1, 324) = 0.06, *p* = .808, η*_p_*^2^ < .001, nor between statements with “true” feedback (*M*_true_ = .74) and statements with “false” feedback (*M*_false_ = .71), *F*(1, 324) = 3.84, *p* = .051, η*_p_*^2^ = .012.

#### Exploratory analysis of SIMs (not preregistered)

We had preregistered to use the CSIM as a measure of correct feedback attributions because it is a well-established measure in research on source memory (Bröder & Meiser, 2007) and memory for truth and falsity (e.g., Nadarevic & Bell, 2024; Nadarevic & Erdfelder, 2019). Importantly, because the CSIM is the proportion of correct source/feedback attributions among the target items correctly identified as old, the measure is less biased by participants’ item memory than the closely related *source identification measure* (SIM) which is the proportion of correct source/feedback attributions among *all* target items (Bröder & Meiser, 2007). However, the problem with our choice of CSIM is that it diverges from Vorms et al. (2022) measure. The authors specified correct feedback attributions as “classification of a true statement as true or a false statement as false” (p.4), which corresponds to the SIM. Thus, we may not have been able to replicate the data pattern of Vorms et al. in the above analysis because we analysed CSIMs instead of SIMs. In fact, when conducting the 2 (plausibility: plausible vs. implausible) × 2 (feedback: “true” vs. “false”) repeated measures ANOVA for SIMs, we found the predicted interaction effect between statement plausibility and veracity feedback, *F*(1, 327) = 7.94, *p* = .005, η*_p_*^2^ = .024 (see Figure 2, right panel), and no significant main effects, *F*s(1, 327) ≤ 2.48, *p*s ≥ .116, η*_p_*^2^s ≤ .008. Pairwise comparisons showed that plausible true statements were more often correctly identified as true than plausible false statements, *t*(327) = 3.03, *p* = .003. For implausible statements, there was a descriptive trend in the opposite direction that was not significant, *t*(327) = 0.74, *p* = .459. These results fully replicate the previous results of Vorms and colleagues.^5^

### Multinomial model analyses

We conducted the multinomial processing tree analyses using the R-package MPTinR (Singmann & Kellen, 2013) to test our hypotheses on effects of statement plausibility on statement memory, feedback memory, and guessing processes.^6^ To obtain an identifiable baseline model, *D*-parameters for new statements and statements without veracity feedback (i.e., “?” feedback) were set equal within each plausibility condition. Equivalent model constraints have been successfully validated (Bayen et al., 1996) and applied in previous research (e.g., Nadarevic & Bell, 2024; Nadarevic & Erdfelder, 2013, 2019). As a goodness-of-fit indicator for our model, we used the likelihood-ratio statistic *G^2^* (Hu & Batchelder, 1994), which is commonly used in MPT modelling. Given the large amount of data on which our model test is based (15,744 data points), the model test was extremely sensitive to even detect tiny deviations at a conventional significance level. For this reason, we set the significance level of the model-fit test to α = .001, which still guarantees high power (1 – β > .99) to detect small model deviations of *w* = .05. The goodness-of-fit test did not display a significant discrepancy between observed and expected response frequencies, *G^2^*(2) = 7.94, *p* = .019, meaning that the base model fit the data. Hypotheses on the model’s parameters were tested by comparing nested models with parameter restrictions against the baseline model. Parameter constraints were tested with Δ*G^2^*-tests at a significance level of α = .05. Parameter tests conducted in line with the preregistered analysis plan are listed in Table 1 and will be explained in more detail in the following sections.

**Table 1:** Conducted parameter tests.


TEST	DESCRIPTION	TESTED PARAMETER CONSTRAINTS

1	Test of Hypothesis 1	*D*_P,true_ = *D*_P,false_ , *D*_I,true_ = *D*_I,false_

2a	Test of Hypothesis 2	*d*_P,true_ = *d*_P,false_ , *d*_I,true_ = *d*_I,false_

2b	Post-hoc test	*s*_P,d_ = *s*_I,d_

3	Test of Hypothesis 3	*a*_P,true_ = *a*_I,true_ , *g*_P,true_ = *g*_I,true_


*Note*. The parameters represent statement memory (*D*), feedback memory (*d*), parameter shrinkage (*s*), and different guessing processes (*a* and *g)*. The parameter index P refers to plausible statements and I to implausible statements.

#### Preregistered analysis of memory parameters

**Figure 3:**
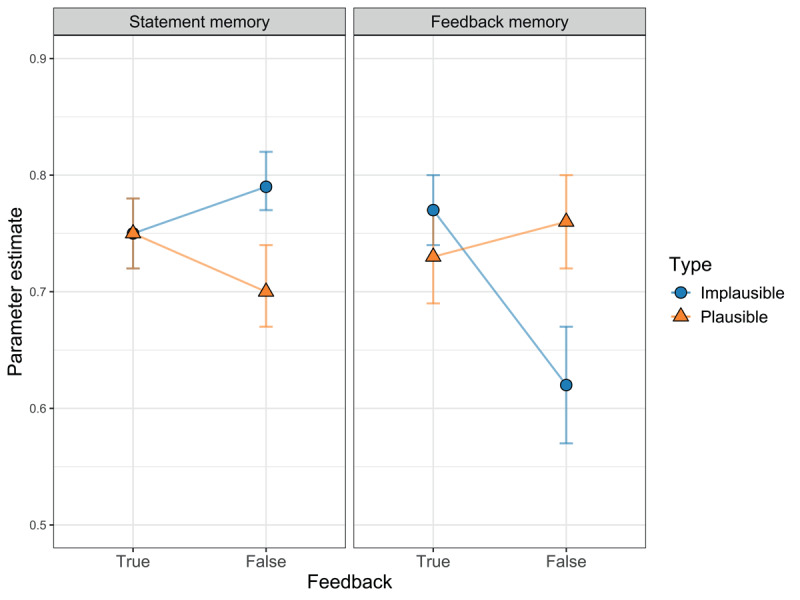
Parameter estimates with 95% confidence intervals for statement memory (*D*) and feedback memory (*d*), depending on statement type (plausible vs. implausible) and veracity feedback (true vs. false).

We predicted an expectation-violation effect on statement memory (hypothesis 1) and feedback memory (hypothesis 2), which means that for plausible statements we expected “false” statements and “false” feedback to be remembered better than “true” statements and “true” feedback, whereas for implausible statements we expected the reverse pattern. To test for this interaction, we first tested if equating *D*-parameters and *d*-parameters, respectively, between statements with “true” and “false” feedback within each plausibility condition would significantly reduce model fit (see Tests 1 and 2a in Table 1). In fact, this was the case for statement memory, Δ*G^2^*(2) = 11.05, *p* = .004, as well as for feedback memory, Δ*G^2^*(2) = 32.58, *p* < .001. However, an inspection of the descriptive pattern of parameter estimates revealed that only the pattern of feedback-memory parameters (*d*) matched the predicted expectation-violation effect. That is, for implausible statements, “true” feedback was remembered better than “false” feedback, and for plausible statements, it was remembered slightly worse than “false” feedback. In contrast, statement-memory parameters (*D*) showed the opposite pattern (see Figure 3). Here, implausible statements were better remembered when presented with “false” feedback than with “true” feedback, while plausible statements were remembered better when presented with “true” feedback than “false” feedback. Estimates for all statement-memory parameters and feedback-memory parameters are reported in Table 2.

**Table 2:** Parameters for statement memory (*D*) and feedback memory (*d*) with 95% confidence intervals for each statement type and feedback.


TYPE	STATEMENT MEMORY			FEEDBACK MEMORY		
	
	*D_true_*	*D_false_*	*D_?_*	*d_true_*	*d_false_*	*d_?_*

plausible	.75 [.72, .78]	.70 [.67, .74]	.70 [.68, .72]	.73 [.69, .77]	.76 [.72, .80]	.23 [.09, .36]

implausible	.75 [.72, .78]	.79 [.77, .82]	.72 [.70, .74]	.77 [.74, .80]	.62 [.57, .67]	.43 [.36, .50]


As preregistered, we proceeded by examining the observed expectation-violation effect for feedback memory in further detail. Specifically, we compared the magnitude of the effect between plausible and implausible statements. We did so employing a reparametrised model with parametric order constraints on the feedback-memory parameter *d* (Knapp & Batchelder, 2004; Kuhlmann et al., 2019). This reparametrised model contained shrinkage parameters *s* reflecting the ratio of *d*-parameters for expectation-consistent and expectation-inconsistent veracity feedback. Hence, the *s*-parameters allow quantifying the magnitude of the expectation-violation effect in each plausibility condition, with smaller *s*-parameters indicating larger effects. We compared these *s*-parameters between plausible and implausible statements to test for a possible asymmetry of the expectation-violation effect (see Test 2b in Table 1). This test indicated that the expectation-violation effect on the *d*-parameter was stronger for implausible statements (*s*_I,d_ = .80) as compared to plausible statements (*s*_P,d_ = .97), Δ*G^2^*(1) = 12.66, *p* < .001.

#### Preregistered analysis of guessing parameters

Finally, we hypothesised that people show plausibility-consistent feedback guessing (hypothesis 3). This means that whenever participants guessed that they had received veracity feedback to a statement, their probability of guessing that this feedback was “true” should be higher for plausible than for implausible statements. For recognised statements (i.e., statements detected as old), this probability is represented by parameter *a*_true_; for unrecognised statements (i.e., statements guessed as old), this probability is represented by parameter *g*_true_. Thus, restricting *a*_true_-parameters and *g*_true_-parameters between plausibility conditions should significantly reduce model fit if our hypothesis holds (see Test 3 in Table 1). In fact, this was the case, Δ*G^2^*(2) = 127.58, *p* < .001. Importantly, as expected, the tendency to guess that alleged veracity feedback was “true” was higher for plausible statements than for implausible statements, irrespective of whether statements were recognised as old (*a*_true_) or not (*g*_true_). Estimates for all guessing parameters are reported in Table 3.

**Table 3:** Guessing parameters with 95% confidence intervals for each statement type.


TYPE	GUESS “OLD”	GUESS “FEEDBACK”		GUESS “TRUE”	
	
	*b*	*a_fb_*	*g_fb_*	*a_true_*	*g_true_*

plausible	.43 [.40, .47]	.44 [.36, .51]	.73 [.68, .78]	.54 [.48, .60]	.65 [.58, .71]

implausible	.37 [.33, .40]	.56 [.50, .61]	.64 [.58, .71]	.29 [.25, .34]	.36 [.28, .44]


## Discussion

The goal of the presented experiment was to 1) test the replicability of the plausibility effect on remembering truth and falsity reported by Vorms et al. (2022) and 2) investigate the cognitive processes underlying this effect. Our results on correct feedback attributions confirmed the plausibility effect described by Vorms et al. (2022): Plausible statements were significantly more often correctly recognised as true than false, whereas implausible statements showed the opposite trend. However, this interaction effect was statistically significant only when we used SIMs instead of CSIMs to measure correct feedback attributions. Moreover, the effect was somewhat smaller than in Vorms and colleagues’ original work.

By analysing the data with a multinomial processing tree model, we were able to gain the following insights into the underlying mechanisms of the effect: As predicted, participants displayed an expectation-consistent guessing bias. That is, they tended to guess the veracity feedback in line with a statement’s plausibility—for plausible statements, they were more likely to guess that the feedback was “true”, whereas for implausible statements, they were more likely to guess that it was “false”. This result occurred regardless of whether they initially recognised the statement in the test phase. This guessing pattern can account for the above-reported plausibility effect on correct feedback attributions. In contrast, feedback memory was better when there was a mismatch between statement plausibility and the provided veracity feedback. This finding aligns with the expectation-violation model (Nadarevic & Bell, 2024), which served as a theoretical framework for this study. Surprisingly, however, we only observed this expectation-violation effect for feedback memory but not for statement memory. In fact, participants were better at remembering false implausible statements than false plausible statements, whereas statement memory did not differ between implausible and plausible true statements.

Notably, the pattern of memory parameters in our study stands in direct opposition to the results of Nadarevic and Bell (2024), who manipulated the base rates of “true” and “false” feedback between participants to induce specific feedback expectations. This context-level manipulation led to an expectation-violation effect for statement memory but had no impact on feedback memory. In the current study, in contrast, expectations varied on the item level—between plausible and implausible statements. Here, we found an expectation-violation effect on feedback memory, but not statement memory. To the contrary, the latter revealed an effect in the opposite direction. These contrasting findings suggest that different mechanisms are at play when manipulating expectations via base rates or plausibility. In base-rate manipulations, no expectations are tied to individual items; instead, statements associated with rare feedback are more salient, potentially explaining the expectation-violation effect on statement memory reported by Nadarevic and Bell. In contrast, a plausibility manipulation induces expectation of veracity on a single-item level, without introducing differences in salience. This can explain the expectation-violation effect on feedback memory found in the present study. What remains unclear, however, is how to account for the expectation-consistency effect found in statement memory for statements presented with “false” feedback.

One limitation of our study is that we pretested the plausibility of the materials, but we did not directly assess the individually perceived plausibility of each participant. The primary reason for this decision was to remain consistent with the original study reporting the plausibility effect (Vorms et al., 2022). In contrast, a study by Niedziałkowska and Nieznański (2021, Experiment 2) investigated feedback memory for moderately plausible statements, and asked participants to judge the veracity of these statements before the study phase. The findings of this study partially align with the current results: when people’s a priori veracity judgment was consistent with the veracity feedback, target recollection (comparable to statement memory) was better than for inconsistent cases. However, for context recollection (comparable to feedback memory), “true” feedback was always remembered better independently of the initial judgement, which contradicts the present findings. Possibly, this discrepancy relates to the degree to which the veracity feedback violated participants’ expectations, which was probably to a lower extent in Niedziałkowska and Nieznański’s study that only used moderately plausible statements. It would thus be valuable to examine the role of surprise in modulating the plausibility effect in future research. A recent study by De Bruïne et al. (2025) found a positive relationship between surprise ratings and memory performance in an immediate recall test, but only for plausible items. No significant effect was observed for implausible items. Following this idea, incorporating surprise ratings (i.e., assessing how unexpected the feedback was) could also be a promising direction for extending the current study. This could also lead to insights into why we did not find symmetrical expectation-violation effects on feedback memory for plausible and implausible statements in our study.

## Conclusion

In this study, we successfully replicated the plausibility effect in remembering truth and falsity, as reported by Vorms et al. (2022). Given the replication crisis in psychological research, this finding is in itself noteworthy. Moreover, by employing a multinomial processing tree model, we could a) demonstrate that the plausibility effect primarily reflects an expectation-consistent guessing bias rather than a genuine memory effect and b) gain new insights into the interplay of statement plausibility and veracity feedback on memory for both statements and feedback. Taken together, our findings highlight that conclusions drawn from potentially confounded measures, such as SIMs and CSIMs, should be interpreted with caution and ideally replaced by methodological approaches that disentangle and quantify distinct cognitive processes.

## Data Accessibility Statement

The materials, the descriptions of the pretest and the power analyses, the programmed experiment, the laboratory log, the data, and the analysis code for the experiment are available at https://osf.io/7gyq4/.

## Footnotes

For example, consider the plausible and false statements from our material: “In Saudi Arabia, it is illegal for women to divorce.” A person may have background knowledge that Saudi Arabia has strict legal regulations concerning women’s rights, but at the same time, the statement is novel to them, and they may not know if it’s actually true or false. Nevertheless, they consider a statement as highly plausible based on their background knowledge.

In research on memory for truth and falsity, cognitive load is usually used to differentiate between two competing memory models: Spinozan and Cartesian (e.g. Ford & Nieznański, 2023; Nadarevic & Erdfelder, 2019). In contrast, our study’s objectives do not require inducing cognitive load.

Note that it is not possible to reanalyse the data of Vorms et al. 2022 with the described MPT model. In their procedure, statements presented without veracity feedback in the learning phase were not included in the final memory test. This means that a statement condition required for fitting the MPT model is missing.

The programmed experiment file is accessible on OSF (https://osf.io/7gyq4/) and can be executed in a web browser.

Note that non-preregistered pairwise comparisons on CSIMs showed the same results. That is, significantly higher CSIMs for plausible true compared to plausible false statements, *t*(324)= 2.38, *p* = .018, but no significant difference in CSIMs between implausible true and implausible false statements, *t*(324)= 0.39, *p* = .695.

Analyses with MPTinR are based on pooled data, i.e., data aggregated across items and participants for each model category. To test the robustness of our results, we aimed at repeating all MPT analyses with a Bayesian latent-trait approach with partial data pooling (Klauer, 2010), using the R-package TreeBUGS (Heck et al., 2018). However, despite using 200,000 iterations in the analysis, the model did not converge, with *R̂* values exceeding 1.05.

## References

[1] Batchelder, W. H., & Riefer, D. M. (1999). Theoretical and empirical review of multinomial process tree modeling. Psychonomic Bulletin & Review, 6(1), 57–86. 10.3758/BF03210812 12199315

[2] Bayen, U. J., & Kuhlmann, B. G. (2011). Influences of source–item contingency and schematic knowledge on source monitoring: Tests of the probability-matching account. Journal of Memory and Language, 64(1), 1–17. 10.1016/j.jml.2010.09.001 21603251 PMC3095109

[3] Bayen, U. J., Murnane, K., & Erdfelder, E. (1996). Source discrimination, item detection, and multinomial models of source monitoring. Journal of Experimental Psychology: Learning, Memory, and Cognition, 22(1), 197–215. 10.1037/0278-7393.22.1.197

[4] Bayen, U. J., Nakamura, G. V., Dupuis, S. E., & Yang, C.-L. (2000). The use of schematic knowledge about sources in source monitoring. Memory & Cognition, 28(3), 480–500. 10.3758/BF03198562 10881564

[5] Bell, R., Buchner, A., & Musch, J. (2010). Enhanced old–new recognition and source memory for faces of cooperators and defectors in a social-dilemma game. Cognition, 117(3), 261–275. 10.1016/j.cognition.2010.08.020 20869699

[6] Bröder, A., & Meiser, T. (2007). Measuring Source Memory. Zeitschrift Für Psychologie/Journal of Psychology, 215(1), 52–60. 10.1027/0044-3409.215.1.52

[7] de Bruïne, A., Vel Tromp, M., Koornneef, A., Brod, G., & Jolles, D. (2025). The interactive effects of surprise and plausibility on memory. Journal of Experimental Psychology: Learning, Memory, and Cognition, 51(6), 954–967. 10.1037/xlm0001388 39666513

[8] Erdfelder, E., Auer, T.-S., Hilbig, B. E., Aßfalg, A., Moshagen, M., & Nadarevic, L. (2009). Multinomial Processing Tree Models: A Review of the Literature. Zeitschrift Für Psychologie/Journal of Psychology, 217(3), 108–124. 10.1027/0044-3409.217.3.108

[9] Faul, F., Erdfelder, E., Lang, A.-G., & Buchner, A. (2007). G*Power 3: A flexible statistical power analysis program for the social, behavioral, and biomedical sciences. Behavior Research Methods, 39(2), 175–191. 10.3758/BF03193146 17695343

[10] Fazio, L. K., & Marsh, E. J. (2009). Surprising feedback improves later memory. Psychonomic Bulletin & Review, 16(1), 88–92. 10.3758/PBR.16.1.88 19145015 PMC4036076

[11] Fazio, L. K., Rand, D. G., & Pennycook, G. (2019). Repetition increases perceived truth equally for plausible and implausible statements. Psychonomic Bulletin & Review, 26(5), 1705–1710. 10.3758/s13423-019-01651-4 31420808

[12] Ford, D., & Nieznański, M. (2023). Cognitive load reduces context recollection for true sentences. Journal of Cognitive Psychology, 35(6–7), 663–676. 10.1080/20445911.2023.2245600

[13] Heck, D. W., Arnold, N. R., & Arnold, D. (2018). TreeBUGS: An R package for hierarchical multinomial-processing-tree modeling. Behavior Research Methods, 50(1), 264–284. 10.3758/s13428-017-0869-7 28374146 PMC5809562

[14] Henninger, F., Shevchenko, Y., Mertens, U., Kieslich, P. J., & Hilbig, B. E. (2024). lab.js: A free, open, online experiment builder (Version v23.0.0-alpha4) [Computer software]. Zenodo. 10.5281/ZENODO.597045

[15] Hu, X., & Batchelder, W. H. (1994). The statistical analysis of general processing tree models with the EM algorithm. Psychometrika, 59(1), 21–47. 10.1007/BF02294263

[16] Keefe, R. S. E., Arnold, M. C., Bayen, U. J., McEvoy, J. P., & Wilson, W. H. (2002). Source-monitoring deficits for self-generated stimuli in schizophrenia: Multinomial modeling of data from three sources. Schizophrenia Research, 57(1), 51–67. 10.1016/S0920-9964(01)00306-1 12165376

[17] Klauer, K. C. (2010). Hierarchical multinomial processing tree model: A latent-trait approach. Psychometrika, 75(1), 70–98. 10.1007/S11336-009-9141-0

[18] Knapp, B. R., & Batchelder, W. H. (2004). Representing parametric order constraints in multi-trial applications of multinomial processing tree models. Journal of Mathematical Psychology, 48(4), 215–229. 10.1016/j.jmp.2004.03.002

[19] Krawczyk, M. C., Fernández, R. S., Pedreira, M. E., & Boccia, M. M. (2017). Toward a better understanding on the role of prediction error on memory processes: From bench to clinic. Neurobiology of Learning and Memory, 142, 13–20. 10.1016/j.nlm.2016.12.011 28017817

[20] Kuhlmann, B. G., Erdfelder, E., & Moshagen, M. (2019). Testing interactions in multinomial processing tree models. Frontiers in Psychology, 10, 2364. 10.3389/fpsyg.2019.02364 31736818 PMC6837999

[21] Lange, K., Kühn, S., & Filevich, E. (2015). “Just Another Tool for Online Studies” (JATOS): An easy solution for setup and management of web servers supporting online studies. PLOS ONE, 10(6), e0130834. 10.1371/journal.pone.0130834 26114751 PMC4482716

[22] Mercier, H. (2017). How gullible are we? A review of the evidence from psychology and social science. Review of General Psychology, 21(2), 103–122. 10.1037/gpr0000111

[23] Moshagen, M. (2010). multiTree: A computer program for the analysis of multinomial processing tree models. Behavior Research Methods, 42(1), 42–54. 10.3758/BRM.42.1.42 20160285

[24] Murnane, K., & Bayen, U. J. (1996). An evaluation of empirical measures of source identification. Memory & Cognition, 24(4), 417–428. 10.3758/BF03200931 8757491

[25] Nadarevic, L. (2025). Effects of statement type and study context on memory for truth and falsity. PsyArXiv. 10.31234/osf.io/9t8fb_v1 42275008

[26] Nadarevic, L., & Bell, R. (2024). Remembering the truth or falsity of advertising claims: A preregistered model-based test of three competing theoretical accounts. Psychonomic Bulletin & Review, 31(5), 2323–2331. 10.3758/s13423-024-02482-8 38528303 PMC11543714

[27] Nadarevic, L., & Erdfelder, E. (2013). Spinoza’s error: Memory for truth and falsity. Memory & Cognition, 41(2), 176–186. 10.3758/s13421-012-0251-z 22972664

[28] Nadarevic, L., & Erdfelder, E. (2019). More evidence against the Spinozan model: Cognitive load diminishes memory for “true” feedback. Memory & Cognition, 47(7), 1386–1400. 10.3758/s13421-019-00940-6 31215012

[29] Niedziałkowska, D., & Nieznański, M. (2021). Recollection of “true” feedback is better than “false” feedback independently of a priori beliefs: An investigation from the perspective of dual-recollection theory. Memory, 29(9), 1186–1196. 10.1080/09658211.2021.1973037 34468262

[30] R Core Team. (2022). R: A language and environment for statistical computing. [Computer software]. https://www.R-project.org/

[31] Richter, T. (2015). Validation and comprehension of text information: Two sides of the same coin. Discourse Processes, 52(5–6), 337–355. 10.1080/0163853X.2015.1025665

[32] Schmidt, O., Erdfelder, E., & Heck, D. W. (2025). How to develop, test, and extend multinomial processing tree models: A tutorial. Psychological Methods, 30(4), 720–743. 10.1037/met0000561 37498691

[33] Singer, M. (2013). Validation in reading comprehension. Current Directions in Psychological Science, 22(5), 361–366. 10.1177/0963721413495236

[34] Singer, M. (2019). Challenges in processes of validation and comprehension. Discourse Processes, 56(5–6), 465–483. 10.1080/0163853X.2019.1598167

[35] Singmann, H., & Kellen, D. (2013). MPTinR: Analysis of multinomial processing tree models in R. Behavior Research Methods, 45(2), 560–575. 10.3758/s13428-012-0259-0 23344733

[36] Snodgrass, J. G., & Corwin, J. (1988). Pragmatics of measuring recognition memory: Applications to dementia and amnesia. Journal of Experimental Psychology: General, 117(1), 34–50. 10.1037/0096-3445.117.1.34 2966230

[37] Vorms, M., Harris, A. J. L., Topf, S., & Hahn, U. (2022). Plausibility matters: A challenge to Gilbert’s “Spinozan” account of belief formation. Cognition, 220, 104990. 10.1016/j.cognition.2021.104990 35026693

[38] Wiswede, D., Koranyi, N., Müller, F., Langner, O., & Rothermund, K. (2013). Validating the truth of propositions: Behavioral and ERP indicators of truth evaluation processes. Social Cognitive and Affective Neuroscience, 8(6), 647–653. 10.1093/scan/nss042 22461436 PMC3739909

[39] Wulff, L., Bell, R., Mieth, L., & Kuhlmann, B. G. (2021). Guess what? Different source-guessing strategies for old versus new information. Memory, 29(3), 416–426. 10.1080/09658211.2021.1900260 33726623

